# Return to work after mild traumatic brain injury: association with positive CT and MRI findings

**DOI:** 10.1007/s00701-022-05244-4

**Published:** 2022-05-31

**Authors:** Antti Huovinen, Ivan Marinkovic, Harri Isokuortti, Antti Korvenoja, Kaisa Mäki, Taina Nybo, Rahul Raj, Susanna Melkas

**Affiliations:** 1 Neurology, University of Helsinki and Helsinki University Hospital (Mr Huovinen, Drs Marinkovic, Isokuortti and Melkas), Haartmaninkatu 4, P.O. Box 340, N00029 Helsinki, HUS Finland; 2 HUS Medical Imaging Center, Radiology, University of Helsinki and Helsinki University Hospital (Dr Korvenoja), Haartmaninkatu 4, P.O. Box 340, N00029 Helsinki, HUS Finland; 3 Neuropsychology, University of Helsinki and Helsinki University Hospital (Ms Mäki and Dr Nybo), Haartmaninkatu 4, P.O. Box 340, N00029 Helsinki, HUS Finland; 4 Neurosurgery, University of Helsinki and Helsinki University Hospital (Dr. Raj), Topeliuksenkatu 5, P.O. Box 266, N00029 Helsinki, HUS Finland

**Keywords:** Mild traumatic brain injury, Return to work, Traumatic intracranial lesions, Functional recovery, Post-concussion symptoms

## Abstract

**Background:**

Return to work (RTW) might be delayed in patients with complicated mild traumatic brain injury (MTBI), i.e., MTBI patients with associated traumatic intracranial lesions. However, the effect of different types of lesions on RTW has not studied before. We investigated whether traumatic intracranial lesions detected by CT and MRI are associated with return to work and post-concussion symptoms in patients with MTBI.

**Methods:**

We prospectively followed up 113 adult patients with MTBI that underwent a brain MRI within 3–17 days after injury. Return to work was assessed with one-day accuracy up to one year after injury. Rivermead Post-Concussion Symptoms Questionnaire (RPQ) and Glasgow Outcome Scale Extended (GOS-E) were conducted one month after injury. A Kaplan–Meier log-rank analysis was performed to analyze the differences in RTW.

**Results:**

Full RTW-% one year after injury was 98%. There were 38 patients with complicated MTBI, who had delayed median RTW compared to uncomplicated MTBI group (17 vs. 6 days), and more post-concussion symptoms (median RPQ 12.0 vs. 6.5). Further, RTW was more delayed in patients with multiple types of traumatic intracranial lesions visible in MRI (31 days, *n* = 19) and when lesions were detected in the primary CT (31 days, *n* = 24). There were no significant differences in GOS-E.

**Conclusions:**

The imaging results that were most clearly associated with delayed RTW were positive primary CT and multiple types of lesions in MRI. RTW-% of patients with MTBI was excellent and a single intracranial lesion does not seem to be a predictive factor of disability to work.

## Introduction

Traumatic intracranial lesions in mild traumatic brain injury (MTBI) are common and are associated with use of antithrombotic drugs and advancing age [7, 13, 18, 20]. In MTBI, lesions are seldom life-threatening and patients with complicated MTBI rarely need follow-up scans or neurosurgical intervention [7, 13, 20, 28, 36]. Recovery after complicated MTBI is related to various factors such as lesion type and location, age, level of education and psychiatric profile [35, 39].

Return to work (RTW), an important outcome parameter, might be delayed in patients with complicated MTBI [14]. Still, MTBI is not considered a long-term risk factor for disability to work [2].

In most cases, full recovery is expected, even though a minority has been reported to experience persistent post-concussion symptoms [6, 12, 17, 33].

Types and locations of intracranial lesions in MTBI have been well characterized [13], but to our knowledge, the influence of lesions on RTW in MTBI has not been studied before. In addition, the influence of primary CT finding is of interest, since CT is primary imaging modality in emergency units. Nevertheless, up to one-third of patients with MTBI and a normal head CT have an abnormal MRI [41]. Thus, accounting for traumatic MRI findings is necessary to fully understand the relationship between MTBI and RTW.

The main objective of this study was to compare RTW in complicated and uncomplicated MTBI, and to assess the association between traumatic intracranial lesions and RTW. We hypothesized that overall RTW would be similar between the groups but that RTW would be delayed in MTBI patients with traumatic CT and/or MRI findings (i.e., complicated MTBI) in comparison with MTBI patients with negative CT and/or MRI findings (i.e., uncomplicated MTBI).

## Methods

### Patients

Our prospective cohort included 131 consecutive patients with MTBI (age 18–68 years) from the Traumatic Brain Injury Outpatient Clinic of Helsinki University Hospital. Patients from the catchment area of the Helsinki University Hospital, home to 2 million inhabitants, were prospectively recruited from 2015 to 2018 and evaluated in the clinic one month after injury.

In this study, patients with alcohol or drug dependence were excluded. Dependence was defined according to the Diagnostic and Statistical Manual of Mental Disorders, 4th edition (DSM-IV) criteria. In addition, visual or auditory disability, previously diagnosed schizophrenia or schizoaffective disorder, contraindications for MRI imaging and not being a native Finnish speaker were also exclusion criteria. For this study, we also excluded patients who were not employed during the time of injury, and patients who underwent MRI imaging later than 17 days after injury. This resulted in a total of 113 patients diagnosed with MTBI.

All included patients gave their written consent. This study was additionally approved by the ethics committee of Helsinki University Hospital (code 105/13/03/01/2014).

### MTBI classification

We used the World Health Organization (WHO) definition of MTBI [3]. These criteria include one or more of the following: 1) confusion or disorientation, loss of consciousness for 30 min or less, post-traumatic amnesia for less than 24 h and/or other transient neurological abnormalities such as focal signs, seizure and intracranial lesion not requiring surgery; and 2) Glasgow Coma Scale score of 13–15 after 30 min post-injury or later upon presentation for healthcare. These manifestations of MTBI could not be due to drugs, alcohol, medications, caused by other injuries or treatment for other injuries (e.g., systemic injuries, facial injuries or intubation), caused by other problems (e.g., psychological trauma, language barrier or coexisting medical conditions), or caused by penetrating craniocerebral injury. MTBI was defined as complicated when a traumatic intracranial abnormality was present in CT or MRI imaging. Isolated skull fractures were not considered as complicated MTBI.

### Initial evaluation

Patients with MTBI were initially evaluated in the Helsinki University Hospital or city hospital emergency units. Initial GCS, loss of consciousness (LOC) and presence and length of post-traumatic amnesia (PTA) along with relevant clinical findings were documented by physicians in the emergency unit, who also determined the initial sick leave length. At this point, patients with more severe TBI were excluded. Primary CT was obtained from 106 (94%) of the patients. Acute symptoms, such as headache and vomiting, were screened and other injuries were determined by Abbreviated Injury Scale and Injury Severity Score, retrospectively from hospital records [5].

At one month after injury, patients were evaluated by a board certified neurologist in the Traumatic Brain Injury Outpatient Clinic of Helsinki University Hospital, using the Neurological Outcome Scale for TBI (NOS-TBI) [37]. Previous and current illnesses and medications were thoroughly assessed using hospital records and by conducting a structured interview. Successful RTW was verified and time to RTW was documented. When deemed requisite, sick leave was extended and additional appointments were arranged in addition to the research protocol.

Additionally, at one month after injury, the presence of post-traumatic symptoms was assessed using the Rivermead Post-Concussion Symptoms Questionnaire (RPQ), evaluating the frequency and severity of 16 post-concussion symptoms, including various physical, emotional and cognitive symptoms [19]. Overall recovery assessed using Glasgow Outcome Scale Extended (GOS-E) [16]. GOS-E is an assessment tool for functional recovery, a scale ranging from 1 (dead) to 8 (good recovery). A score of ≥ 6 is considered as a favorable outcome for patients with TBI [32]. Patients, who have fully returned to work, are generally considered GOS-E = 8, though some patients may work with minor post-concussion symptoms [31].

### Return to work evaluation

Return to work was assessed retrospectively with one-day accuracy using clinic records, and patients’ successful RTW was later verified by a structured telephone interview at one year after injury, by the study author (AH). In Finland, the sick leaves are registered electronically thus considered reliable [34]. Thus, RTW could be documented with one-day accuracy. Full RTW was determined days from injury to the first day back to full-time work, with no further significant sick leave in the follow-up period. Patients were not considered to have fully returned to work until their possible partial labor period was over. Patients who were full-time (*n* = 13) or part-time (*n* = 6) students were also included in this study and their return to studies was comparable and included in RTW parameters.

RTW was assessed as a continuous variable (time to RTW) with one-day accuracy and dichotomously (RTW-%) with predetermined cutoff points: 14 days, 30 days, 90 days and one year after injury.

### Brain imaging

All patients underwent brain MRI imaging within 3–17 days (mean 9.6, SD 3.2) after MTBI and all MRI scans were evaluated by a board certified neuroradiologist. Lesions were assessed systematically by using common data elements (CDE) for TBI neuroimaging [11].

All imaging was performed with 3 T Siemens Magnetom Verio (Siemens, Erlangen) scanner with a 32-channel head coil. The imaging protocol consisted of fast localizer, T1 sagittal localizer, axial FLAIR, coronal T2, 3D T2 SPACE, 3D T1 MPRAGE and 3D gradient-echo susceptibility weighted imaging (SWI) sequence.

Investigated traumatic intracranial lesions included extra-axial lesions (subdural hemorrhages [SDH], subarachnoid hemorrhages [SAH] and epidural hemorrhages [EDH]) and intraparenchymal lesions (cerebral contusions, traumatic microbleeds [TMBs] and other intracerebral hemorrhages [ICH]). Traumatic microbleeds were defined as a single or several small hemorrhagic lesion(s) in the white matter or grey–white interface, detected with SWI sequence. No non-hemorrhagic diffuse axonal injury lesions were detected. The presence of each of the lesions was assessed. The location or the number of lesions were not specified in this study.

**Fig. 1:**
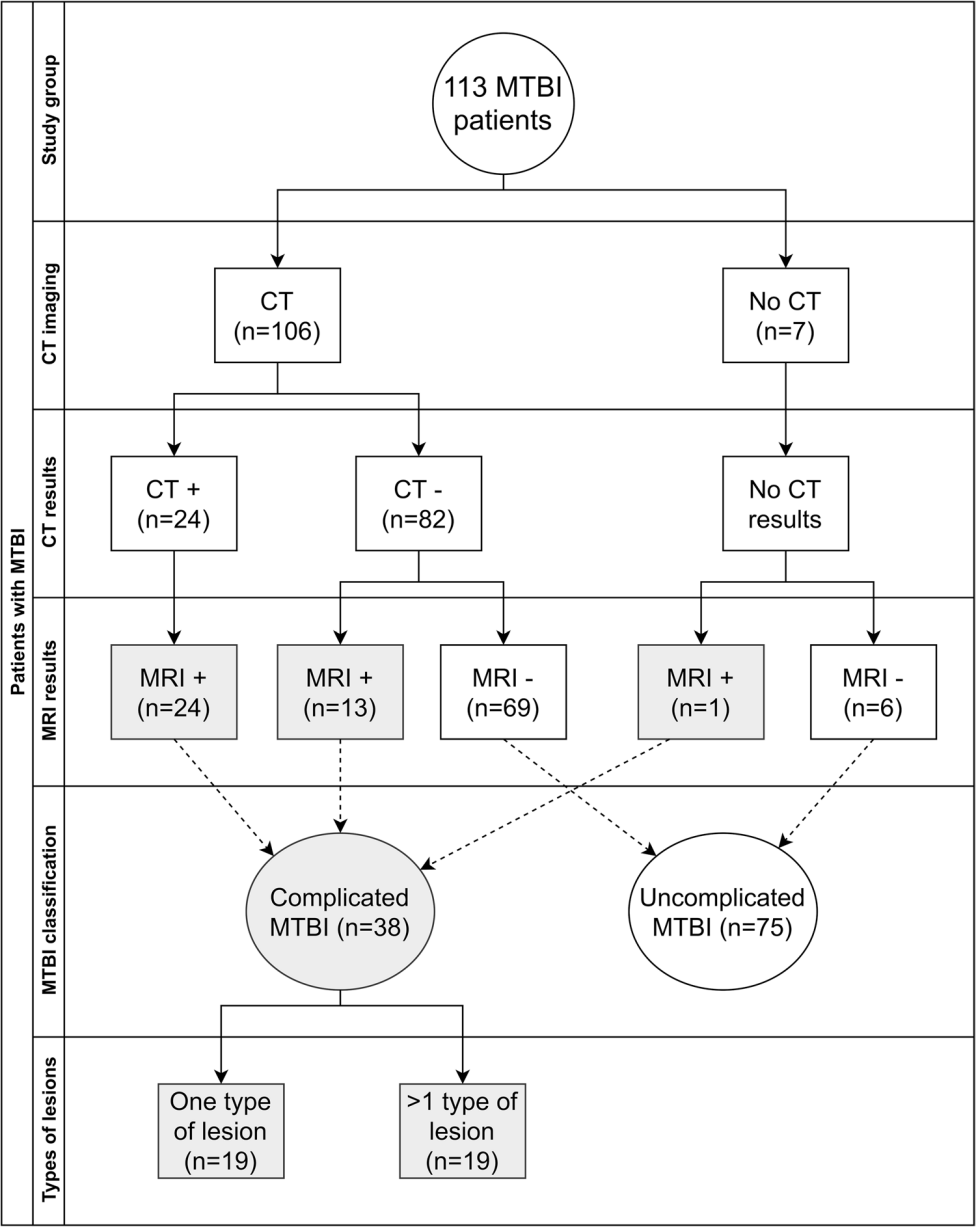
Distribution of CT and MRI findings in the study group

Differentiation between the patients with uncomplicated MTBI (*n* = 75) and those with complicated MTBI (*n* = 38) was based on the findings in the conventional 3 T MRI, including SWI sequences. The latter group was furthermore divided into those who had only one type of traumatic intracranial lesion (*n* = 19), and those who had more than one type of traumatic intracranial lesions (*n* = 19). Additionally, we analyzed following subgroups separately: patients who did not undergo primary CT imaging (*n* = 7), patients with no traumatic intracranial lesions visible in the primary CT (*n* = 82) and patients with one or more traumatic intracranial lesions visible in the primary CT (*n* = 24). Figure 1 shows a flowchart on the relation between findings in CT and MRI.

### Statistical analysis

Skewed distributions were reported as medians with interquartile range (IQR) and normally distributed values in mean and standard deviation (SD). Skewed data were compared between groups using a nonparametric Mann–Whitney U test. With multiple groups, we used Kruskal–Wallis H test. Categorical variables were compared using a two-sided χ^2^ test (Pearson Chi-square test). Spearman correlation test was used to compare correlation between two continuous variables. A Kaplan–Meier log-rank analysis was performed to investigate the time differences in RTW between groups. We considered p values < 0.05 as statistically significant.

IBM SPSS Statistics 25 (IBM Corp., Armonk, NY, USA) was used to perform the analyses.

### Results

There were 38 patients with complicated MTBI and 24 patients had a traumatic lesion visible in the primary head CT scan. Nineteen patients had more than one type of lesion in MRI, for instance, traumatic microbleeds and SDH or SAH (Fig. 1). The most common mechanism of injury was ground level fall (28%), followed by bicycle accident (26%) and fall from heights (20%).

Median RTW was 9 days (IQR 4.0–30.0). Fifty-seven percent of the patients had full RTW before MRI. RTW-% was 92% at three months and 98% one year after injury. One patient was still working partially and only one patient had not returned to work at all at one year after injury. For all patients, RPQ showed a median of 8.0 points (IQR 3.0–15.0), while in GOS-E, 56% of patients had a good (GOS-E = 8) functional recovery.

There were 14 patients who had traumatic intracranial lesions detected only in the subacute MRI and not in CT (CT-/MRI + group). Of them, 11 had traumatic microbleeds, one had a cerebral contusion, one had SDH, and one patient had both SDH and SAH. Their median RTW was 9 days (IQR 5.0–15.0), median RPQ 10.0 points (IQR 3.5–15.0) and 62% of patients had a good (GOS-E = 8) functional recovery, at one month after injury.

### Complicated vs. uncomplicated MTBI

Clinical characteristics of patients with uncomplicated vs. complicated MTBI are shown in Table 1. There was a significant difference between the groups in length of hospitalization (p = 0.012) and frequency and severity of extracranial injuries (p = 0.024).

**Table 1:** Comparison of clinical characteristics and outcome measures, uncomplicated vs. complicated MTBI

	Uncomplicated MTBI (*n* = 75)			Complicated MTBI (*n *= 38)			p value**
	Valid n	Median/n	SD/%/IQR	Valid n	Median/n	SD/%/IQR	
Age (mean)	75	38.0	12.0	38	41.5	12.6	0.169
Gender (female)	75	37	49.3%	38	12	31.6%	0.072
Years of education (median)	75	16.5	13.0–19.0	38	15.5	12.5–18.0	0.588
Previous or current illness*	75	35	46.7%	38	13	34.2%	0.206
Anticoagulation	75	2	2.7%	38	1	2.6%	0.991
Hospitalization period (days)	75	1.0	1.0–2.0	38	2.0	1.0–2.0	0.012
Loss of consciousness (witnessed)	75	39	52.0%	38	23	60.5%	0.390
Time (minutes)	39	1	1–2	23	1	1–3	0.832
Post-traumatic amnesia	75	70	93.3%	38	34	89.5%	0.474
Time (h:min)	70	1:00	0:20–4:00	34	1:17	0:18–3:00	0.920
GCS measured by first aid	75	40	53.3%	38	21	55.3%	0.846
GCS < 15	40	11	27.5%	21	5	23.8%	0.756
Extracranial Injury Severity Score	75	1	0–2	38	2	1–5	0.024
Return to work, days (median)	75	6	3.0–16.0	38	17	9.5–50.5	< 0.001
RTW-% 14 days after MTBI	75	56	74.7%	38	14	36.8%	< 0.001
RTW-% 30 days after MTBI	75	64	85.3%	38	23	60.5%	0.003
RTW-% 3 months after MTBI	75	71	94.7%	38	33	86.8%	0.147
RTW-% one year after MTBI	75	75	100%	38	36	94.7%	0.045
RPQ points one month post-injury	66	6.5	2.0–13.3	31	12.0	5.0–15.0	0.025
GOS-E = 8, one month post-injury	68	40	58.8%	32	16	50.0%	0.407

^*^Previous or current illnesses include cardiovascular diseases, diabetes and a variety of neurological and psychiatric conditions. No single condition stood out to be significantly more frequent between groups

^**^Categorical variables were compared using a two-sided χ2 test (Pearson Chi-square test). Skewed data were compared between groups using a nonparametric Mann–Whitney U test

**Fig. 2:**
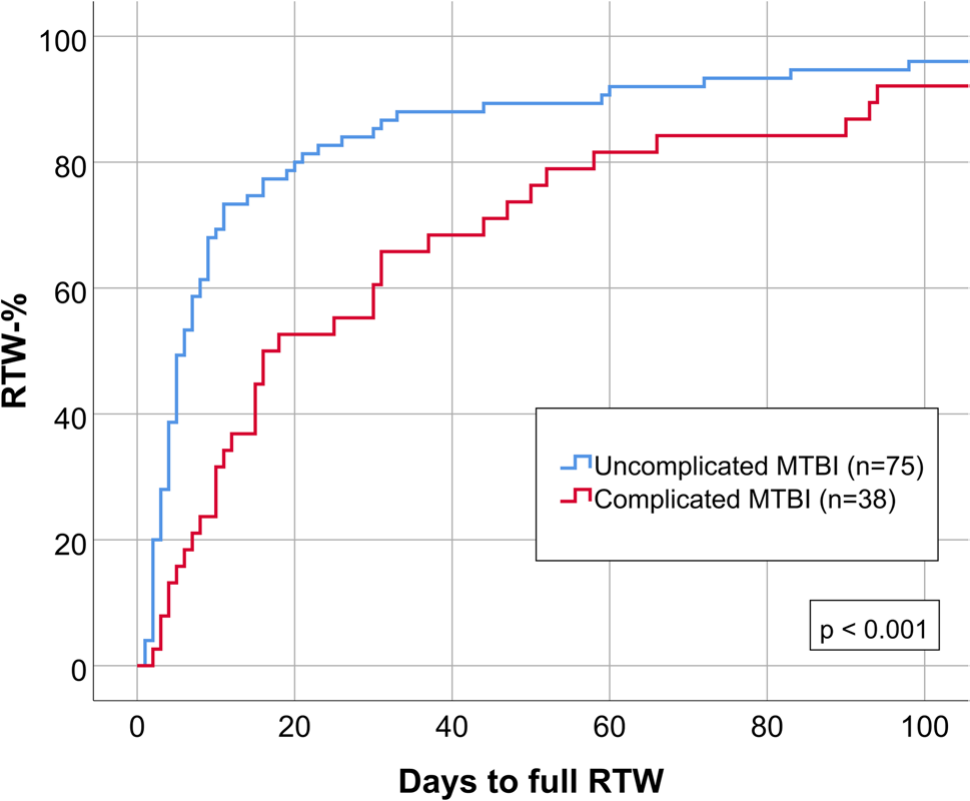
RTW in patients with complicated MTBI (*n* = 38) and patients with uncomplicated MTBI (*n* = 75)

Patients with complicated MTBI had significantly delayed RTW (*n* = 38, median 17 days, IQR 9.5–50.5) compared with patients with uncomplicated MTBI (*n* = 75, median 6 days, IQR 3.0–16.0; p < 0.001), as illustrated in Table 1 and Fig. 2. The most noticeable difference in RTW-% was at 14 days after injury (37% vs. 75%; p < 0.001). There were no significant differences at three months after injury, but RTW-% was lower in the complicated group one year after injury (95% vs. 100%, p = 0.045).

Patients with complicated MTBI (Table 1) suffered significantly more frequently from post-concussion symptoms (median RPQ 12.0, IQR 5.0–15.0) than patients with uncomplicated MTBI (median RPQ 6.5, IQR 2.0–13.3; p = 0.025). There was no significant difference in GOS-E results; 50% vs. 59% had a good functional recovery (GOS-E = 8).

### Positive vs. negative primary CT

**Fig. 3:**
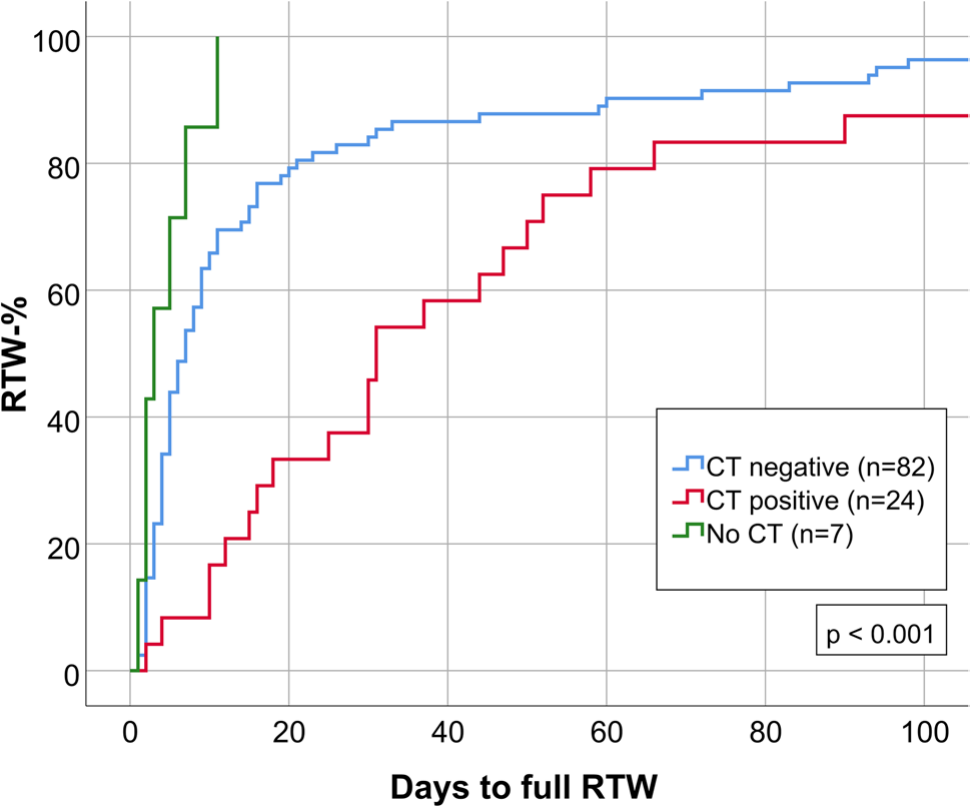
RTW in three subgroups of MTBI regarding CT imaging: patients who did not undergo CT imaging (*n* = 7), patients with CT negative imaging (*n* = 82) and patients with intracranial lesion(s) in primary CT (*n* = 24)

As illustrated in Fig. 3, MTBI patients with CT positive traumatic intracranial lesion(s) had delayed RTW (*n* = 24, median 31 days, IQR 15.3–56.5) compared to patients with negative primary CT (*n* = 82, median 7 days, IQR 4.0–16.0) and those who did not undergo CT imaging (*n* = 7, median 3 days, IQR 2.0–7.0; p < 0.001).

As shown in Table 2, MTBI patients with CT positive traumatic intracranial lesions had post-concussion symptoms more frequently (median RPQ 13.5, IQR 9.3–15.3) than patients with negative primary CT (median RPQ 7.0, IQR 2.0–14.5) and those who did not undergo CT imaging (median RPQ 4.0, IQR 0–10.0; p = 0.017). There were no significant differences in GOS-E (p = 0.186).

**Table 2:** Main outcomes in three subgroups of MTBI regarding CT imaging: patients who did not undergo CT imaging (*n* = 7), patients with CT negative imaging (*n* = 82) and patients with intracranial lesion(s) in primary CT (*n* = 24)

	No CT (*n* = 7)			CT Negative (*n* = 82)			CT positive (*n* = 24)			P value*
	Valid n	Median/n	IQR/%	Valid n	Median/n	IQR/%	Valid n	Median/n	IQR/%	
RTW (days)	7	3.0	2.0–7.0	82	7.0	4.0–16.0	24	31.0	15.3–56.5	< 0.001
RTW-% 14 days after MTBI	7	7	100%	82	58	70.7%	24	5	20.8%	< 0.001
RTW-% 30 days after MTBI	7	7	100%	82	69	84.1%	24	11	45.8%	< 0.001
RTW-% 3 months after MTBI	7	7	100%	82	76	92.7%	24	21	87.5%	0.515
RTW-% one year after MTBI	7	7	100%	82	82	100%	24	22	91.7%	0.023
RPQ points	6	4.0	0–10.0	73	7.0	2.0–14.5	18	13.5	9.3–15.3	0.017
GOS-E = 8	6	5	83.3%	75	43	57.3%	19	8	42.1%	0.186

^*^Categorical variables were compared using a two-sided χ2 test (Pearson Chi-square test). Skewed data were compared between groups using a nonparametric Kruskal–Wallis test

### Influence of number and type of lesions

**Fig. 4:**
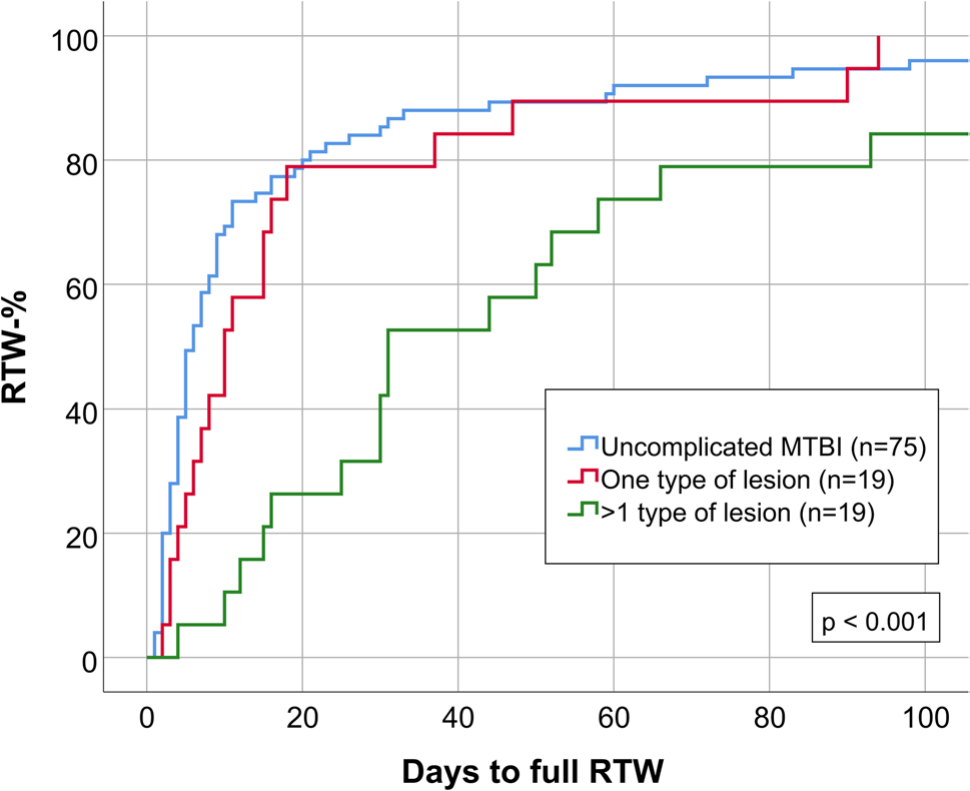
RTW in three subgroups of MTBI: patients with uncomplicated MTBI (*n* = 75): patients with one type of traumatic intracranial lesion (*n* = 19) and patients with more than one types of traumatic intracranial lesions (*n* = 19)

Patients with more than one type of traumatic intracranial lesion had delayed RTW (*n* = 19, median 31 days), compared to those with only one type of lesion (*n* = 19, median 10 days) and patients with uncomplicated MTBI (*n* = 75, median 6 days; p < 0.001), as illustrated in Fig. 4. There were no statistically significant differences in RPQ or GOS-E (Table 3).

**Table 3:** Main outcomes in three subgroups of MTBI: patients with uncomplicated MTBI (*n* = 75): those with one type of traumatic intracranial lesion (*n* = 19) and those with more than one type of traumatic intracranial lesion (*n* = 19) visible in MRI

	Uncomplicated MTBI (*n* = 75)			One type of lesion (*n* = 19)			Multiple types of lesions (*n* = 19)			P value*
	Valid n	Median/n	IQR/%	Valid n	Median/n	IQR/%	Valid n	Median/n	IQR/%	
RTW (days)	75	6.0	3.0–16.0	19	10.0	5.0–18.0	19	31.0	16.0–66.0	< 0.001
RTW-% 14 days after MTBI	75	56	74.7%	19	11	57.9%	19	3	15.8%	< 0.001
RTW-% 30 days after MTBI	75	64	85.3%	19	15	78.9%	19	8	42.1%	< 0.001
RTW-% 3 months after MTBI	75	71	94.7%	19	18	94.7%	19	15	78.9%	0.069
RTW-% one year after MTBI	75	75	100%	19	19	100%	19	17	89.5%	0.006
RPQ points	66	6.5	2.0–13.3	16	11.0	4.3–14.8	15	13.0	7.0–16.0	0.067
GOS-E = 8	68	40	58.8%	16	9	56.3%	16	7	43.8%	0.550

^*^Categorical variables were compared using a two-sided χ2 test (Pearson Chi-square test). Skewed data were compared between groups using a nonparametric Kruskal–Wallis test

Regarding specific lesions (Table 4), median RTW was delayed among patients with SDH (median 37.5 days, p < 0.001), SAH (median 33.5 days, p = 0.001) and cerebral contusions (median 50.0 days; p < 0.001), compared to patients without that specific lesion.

**Table 4:** RTW outcomes regarding specific traumatic intracranial lesions

		Median RTW (days)			RTW-% 14 days after MTBI			RTW-% 30 days after MTBI			RTW-% 3 months after MTBI			RTW-% one year after MTBI		
	Valid n*	RTW	IQR	P**	n	%	P**	n	%	P**	n	%	P**	n	%	P**
Uncomplicated MTBI	75	6.0	3.0–16.0		56	74.7%		64	85.3%		71	94.7%		75	100%	
SDH	16	37.5	15.3–92.3	< 0.001	3	18.8%	< 0.001	7	43.8%	0.001	12	75.0%	0.007	14	87.5%	< 0.001
SAH	14	33.5	14.3–54.0	0.001	3	21.4%	0.001	7	50.0%	0.010	12	85.7%	0.351	13	92.9%	0.103
Contusion	11	50.0	16.0–335.0	< 0.001	0	0%	< 0.001	5	45.5%	0.009	8	72.7%	0.013	9	81.8%	< 0.001
Traumatic microbleeds	22	13.5	6.8–31.0	0.063	11	50.0%	0.198	16	72.7%	0.596	20	90.9%	0.828	22	100%	0.483
Other*** intracranial hemorrhages	4	20.5	10.0–31.0	0.308	2	50.0%	0.616	2	50.0%	0.192	3	75.0%	0.200	3	75.0%	< 0.001

^*^Due to possibility of having more than one types of traumatic intracranial lesions simultaneously, one patient could be categorized into multiple categories. Categorical comparisons and their respective p values were calculated between ‘patients with specific lesion vs. no specific lesion,’ not the uncomplicated MTBI baseline

^**^Categorical variables were compared using a two-sided χ2 test (Pearson Chi-square test). Skewed data were compared between groups using a nonparametric Mann–Whitney U test

^***^Three intracerebral hemorrhages and one epidural hemorrhage

Cerebral contusions showed a borderline association with more frequent post-concussion symptoms (median RPQ 14.5, IQR 8.3–21.0; p = 0.049). Regarding other lesions, there were no significant differences in RPQ or GOS-E.

One year after injury, two patients with three types of traumatic intracranial lesions, had not fully returned to work (Tables 1, 2, 3 and 4). RTW-% at one year was significantly lower in the following groups: those with CT positive lesions (92%, p = 0.023), those with more than one type of intracranial lesion (90%, p = 0.006) and those with SDH (88%, p < 0.001), cerebral contusions (82%, p < 0.001) or other intracranial hemorrhages (75%, p < 0.001), compared to other patients with MTBI.

There was a significant but weak positive correlation between RTW and RPQ (0.256, p = 0.011). Fully recovered patients (GOS-E = 8) returned to work faster (median RTW 6.0 days, IQR 3.0–11.8) than patients with impaired recovery (GOS-E < 8) (median RTW 17.5 days, IQR 5.3–58.8, p = 0.001).

### Discussion

In this well-characterized group of patients with MTBI, the imaging results that were most clearly associated with delayed RTW were positive primary CT and finding of more than one type of lesion in MRI. The difference is most noticeable in the first weeks after injury but tend to even out after three months, though a minority of patients have impaired recovery after this. Delayed RTW may result from prolonged symptoms and slower recovery, but also from primary care physicians’ tendency to give longer sick leave when intracranial lesion is visible in the primary CT scan. Nevertheless, RTW-% of patients with MTBI was excellent and a single intracranial lesion does not seem to be a predictive factor of disability to work.

According to meta-analysis by Bloom and colleagues, RTW after MTBI generally varies between 13 and 93 days, and approximately 89% of patients return to work in one year after injury [1]. Our patients returned to work reasonably fast, with a median of 9 days and 98% were fully returned to work by the end of the year. Similar results were found by Wäljas and colleagues, with 97% RTW rate one year after injury [34].

Delayed RTW in patients with complicated MTBI has been previously documented [14, 34], but there is also documentation on that the prognosis does not differ much between uncomplicated and complicated MTBI [2, 6, 17]. Iverson and colleagues found that MTBI patients with intracranial lesions had significantly delayed RTW (36 vs. 6 days) compared to patients with uncomplicated MTBI [14]. Our results (median RTW 17 vs. 6 days) are in line with these findings.

In our study, CT positive trauma lesions seemed to be strongly associated with delayed RTW (median time to RTW 31 days, *n* = 24). Patients with positive CT findings had more post-concussion symptoms at one month after injury, likely explaining the delayed RTW in this group. Conversely, the 14 patients who had traumatic intracranial lesions detected only in the subacute MRI and not in CT, had a shorter RTW than those with acute positive CT findings, similar to patients with uncomplicated MTBI. Since the majority of the patients in our cohort returned to work before MRI (57%) and most of the patients had successful RTW quickly after imaging, it is unlikely that MRI results had a major role in RTW. In our opinion, subacute MRI might provide additional information when targeted to a subgroup of patients with expected difficulties in RTW.

Furthermore, in a study of 378 patients with MTBI by Skandsen and colleagues, not being triaged for CT was associated with a reduced risk of post-concussion symptoms at three months after injury [27]. In our study, we found accordingly that patients with MTBI, who did not undergo CT imaging, were the fastest to return to work (median 3 days, *n* = 7) and reported post-concussion symptoms least frequently. These patients most likely do not meet the head CT criteria and are generally less symptomatic in the emergency units, thus not requiring longer sick leaves [8].

In our cohort, two patients had not fully returned to work at one year post-injury. One of them was still working partially at this point, and only one patient had not returned to work at all. Both patients had three types of traumatic intracranial lesions, indicating an overall more severe injury. Half of the patients with complicated MTBI had multiple different lesions, and their RTW parameters were worse. After excluding patients with multiple types of lesions, only patients with CT positive lesions had delayed RTW, up to one month after injury. No other RTW parameters showed significant differences at any time point.

The effect of multiple traumatic lesions on outcome in MTBI patients is largely unknown. In a large retrospective cohort by Isokuortti and colleagues, 6.4% of patients with MTBI had more than one type of traumatic intracranial lesion in CT imaging [13]. In a prospective observational study by Sharifuddin and colleagues, having multiple traumatic intracranial lesions (144 of 279 complicated MTBI patients) was an independent risk factor for worse repeat CT, which often lead to a neurosurgical intervention [26]. Patients with multiple traumatic intracranial lesions would highly likely benefit from additional care and adequate information and follow-up.

Regarding specific traumatic intracranial lesions in MTBI, their prevalence was comparable to previous studies [8, 13, 23, 36]. Lesions visible in CT (SDH, SAH and cerebral contusions) were associated with delayed RTW, but only cerebral contusions were associated with persistent post-concussion symptoms at one month after injury. However, the significance was weak in our study. This is consistent with previous studies, some of which have found cerebral contusions as a risk factor for persistent post-concussion symptoms and impaired recovery [30, 39, 41], although some studies point to the opposite [42]. Other lesions have not been considered a clinically significant risk factor from this point of view [7, 15].

None of the parameters affected functional recovery (GOS-E) of the patients. This is also consistent with previous literature and general recovery presentation of patients with MTBI, with other risk factors than traumatic intracranial lesions being more significant for long-term recovery [15, 25, 27, 30, 38].

However, a recent large study by Yuh and colleagues found that MTBI patients with SDH, SAH and/or cerebral contusions had impaired (GOS-E < 8) and unfavorable (GOS-E < 5) recovery up to 1 year after injury [40]. Similar to our results, these particular lesions often coincide. It is noteworthy that the recovery in terms of GOS-E was remarkably worse in the study by Yuh (only 47% of MTBI patients had GOS-E = 8 one year post-injury; respectively, in our cohort, 56% of patients had complete recovery at one month after injury). On the other hand, their patients with MTBI were often (31%) admitted to the intensive care unit even without traumatic intracranial lesions in CT imaging, suggesting a higher degree of injury severity. Patients in our cohort were only monitored up to 24–48 h (median 2 days, IQR 1.0–2.0) in case of complicated MTBI. In general, our patients were quite healthy and recovered well regardless of any examined parameters, although a very small subpopulation (*n* = 2) had impaired recovery.

The presence of even minor traumatic lesion causes inevitable, though mostly unwarranted concern in many patients, as well as in health care professionals [24]. Even though the mentioned lesions are small and clinically related to good prognosis, their presence per se might affect the threshold for recommending sick-leaves thus economically affecting both employer and society [22]. Well-timed RTW, however, promotes the subjective feeling of independence and success, as previously stated by Esbjörnsson and colleagues [10]. Prognosis of MTBI is good nevertheless and early reassuring and educational information is considered beneficial for MTBI recovery [9].

It has been well documented that RTW after MTBI is also affected by extracranial co-trauma, age, occupational factors (such as pre-injury unemployment, lower level of education, limited job independence and decision-making latitude), pre-injury substance and alcohol abuse, and post-concussion symptoms such as nausea or vomiting on hospital admission, fatigue and severe head/bodily pain [2, 5, 15, 29, 34, 38]. In addition, various psychological and psychosocial factors, such as mental health problems, acute psychological stress and perceived injustice, affect outcome after MTBI [4, 21, 35].

Our study has multiple strengths: RTW evaluation was precise with one-day accuracy up to one year after injury. In addition, our comprehensive imaging included both CT and 3 T MRI results and we were able to compare them. The study group was well characterized, including extensive neuropsychological and neuropsychiatric examinations. Finally, due to our particular role as a specialized outpatient clinic for patients with TBI, we are closely able to follow patients’ recovery and return to work after their discharge from emergency units.

We also recognize some limitations: first of all, the number of patients was quite low and did not allow analysis of subgroups with isolated or combined specific lesions or their localization. For instance, due to the low sample size of the CT-/MRI + subgroup, the role of subacute MRI in RTW remains unclear. Significant differences might have been achieved in a larger cohort study. Secondly, the majority of patients had favorable outcome nevertheless. Both of these aspects result in decreased likelihood of achieving statistical significance in any of the examined parameters. Finally, in this study, we focus solely on traumatic intracranial lesions, even though we recognize that outcome after MTBI is multifactorial.

In general, one intracranial lesion in MTBI does not seem to be a long-term predictor of disability to work. RTW after MTBI is undoubtedly multifactorial and dependent of other factors than the lesion itself. This imposes the necessity of further research in order to improve and individualize the care of MTBI patients.

## Abbreviations

CT: Computer tomography
GCS: Glasgow Coma Scale
GOS-E: Glasgow Outcome Scale Extended
IQR: Interquartile range
LOC: Loss of consciousness
MRI: Magnetic resonance imaging
MTBI: Mild traumatic brain injury
PTA: Post-traumatic amnesia
RPQ: Rivermead Post-Concussion Symptoms Questionnaire
RTW: Return to work
SAH: Subarachnoid hemorrhage
SDH: Subdural hemorrhage
TBI: Traumatic brain injury
WHO: World Health Organization
